# Suspected bias in selection criteria of target subpopulation and its validation

**DOI:** 10.1186/s13054-023-04566-8

**Published:** 2023-07-12

**Authors:** Masatoshi Uchida, Kentaro Hayashi

**Affiliations:** Department of Emergency and Critical Care Medicine, Dokkyo Medical University, 880 Kitakobayashi, Mibu-Machi, Shimotsuga-Gun, Tochigi, 321-0293 Japan

Dear Editor,

We read with great interest the recent publication by Osawa et al. regarding heterogeneity in the treatment effects of polymyxin B hemadsorption (PMX-HA) [1]. We consider it to be intriguing and express our respect to the authors for conducting such a meticulous analysis.

However, we have concerns that this study may contain biases. We would like to discuss the two biases of concern.

## Survivor treatment selection bias

The authors used data from a retrospective observational study (JSPETIC-DIC study) to analyze the heterogeneity treatment effects of PMX-HA and suggested that prothrombin time–international normalized ratio (PT-INR) > 1.4 or lactate > 3 mmol/L could serve as criteria for a “targeted subpopulation” that may have greater treatment efficacy with PMX-HA. However, the analytical approach of the authors may give rise to survivor treatment selection bias, and consequently, these criteria may not correctly identify such patients.

Survivor treatment selection bias is where the effect of an intervention is overestimated because of not considering the time difference between the start of observation and the time of intervention [2]. Patients who die early after the start of observation cannot receive the intervention owing to their death and are therefore allocated to the control group, making the intervention group appear to have a higher survival rate. The effect of this bias is brought about by patients who died during the period in which they could have received the intervention, and the impact is greater when there are many such patients in the analyzed population. Moreover, this bias occurs owing to reverse causation between the outcome and the intervention, so adjusting for patient characteristics cannot eliminate it.

If survivor treatment selection bias is present, there will be a tendency to overestimate the effect of the intervention in patients who die early after the start of observation. Consequently, there will appear to be a large difference in mortality rates between the intervention and control groups in a population that is likely to experience early mortality after observation. This creates the illusion that the intervention is significantly more effective in such a population. As a result, the selection criteria for patients who are expected to have a large treatment effect could be influenced by this bias.

In their analysis, the authors appear to have used the variable PMX-HA in the JSEPTIC-DIC study data to create a PMX-HA group and control group. According to the definition of JSEPTIC-DIC study data, the variable PMX-HA was “Yes” if the patient received PMX-HA during the first 7 days after ICU admission, and the start of observation was ICU admission [3]. Although the day of receiving PMX-HA was absent in the data, it could be inferred that not all patients in the PMX-HA group received PMX-HA simultaneously with their ICU admission because of the retrospective nature of that study. Therefore, the results of the study could be affected by survivor treatment selection bias and these criteria may not capture patients who may benefit most from PMX-HA.

## Immortal time bias

The authors applied the aforementioned criteria to a validation cohort derived from the EUPHRATES trial [4] data and reported that PMX-HA significantly improved mortality in the “targeted subpopulation.” However, these results could be influenced by immortal time bias.

Immortal time bias arises when there is a delay between cohort entry and completion of the intervention [5]. In this analysis, the exposure was defined as “the standard regimen of two PMX-HA treatments,” that is, two PMX-HA treatments with a 24-h interval [4]. Consequently, the time frame from cohort entry to the first PMX-HA treatment and the 24-h interval between the first and second PMX-HA treatments constitute immortal time. Essentially, the individuals in the PMX-HA group must survive during this time frame. An analysis that neglects to account for this immortal time could lead to overestimation of the effect of PMX-HA.

The authors simply divided the population according to the presence or absence of the exposure. The Kaplan–Meier curve in their Fig. 3 exhibits no fatalities in the PMX-HA group until day 3, which suggests the presence of immortal time. Additionally, the authors used the Cox proportional hazards model, which does not consider immortal time bias. Therefore, the observed significant improvement in mortality among the PMX-HA group in the targeted subpopulation might be overestimated by immortal time bias. It would be more desirable to analyze the treatment allocation in the EUPHRATES trial as an exposure (intention-to-treat analysis) or to perform an analysis considering immortal time.

The above study addresses an important issue using a novel methodology, but biases may influence the results. Nevertheless, it should be noted that even if these biases are present, they may not significantly impact the conclusions. If the authors provide reanalysis results that address these concerns, it would greatly benefit *Critical Care* readers.

## Data Availability

The original data were derived from the manuscript provided by the journal, and the data from the JSEPTIC-DIC study are publicly available (Sci Data. 2018;5:180243).
